# Programmed death 1 blockade, an Achilles heel for MMR-deficient tumors?

**DOI:** 10.1186/s13045-015-0222-5

**Published:** 2015-11-05

**Authors:** Andy Yingjie Lin, Edward Lin

**Affiliations:** SIR RUN RUN SHAW Hospital, School of Medicine, Zhe-Jiang University, Hangzhou, China; P4 Medicine Institute, Seattle Cancer Care Alliance, Fred Hutchinson Cancer Research Center, University of Washington Medical Center, Seattle, WA 98109 USA

## Abstract

Program death receptor-1 (PD-1) is upregulated in many tumors and in tumor microenvironment, and PD-1 blockade has led to remarkable immune-based anti-tumor responses in across many tumor types. Pembrolizumab, an anti-programmed death 1 checkpoint inhibitor, resulted in a high rate of immune response in 41 patients with previously treated mismatch repair (MMR)-deficient tumor including colorectal cancer but not in MMR-stable tumor with expectant toxicities. Both immune-based progression-free and overall survival are quite promising and correlate with high mutation loads in the tumor. MMR-deficient tumors made up not an insignificant proportion of GI and GU cancers and are found mostly in younger patients who had better prognosis than MMR-stable tumors. However, MMR-deficient tumors do not respond to cytotoxic chemotherapy as these agents may require intact DNA mismatch repair to be effective. MMR deficiency occurred as a result of mutations in defined DNA repair complex mutations or epigenetics modifications and gene upstream of DNA repair complex. PD-1 blockade represents our first successful shot at one of the Achilles heels of this MMR-deficient tumor goliath. Only coordinated attack on all of its Achilles heels and healing mechanisms can this tumor Goliath be brought down to its knees.

Program death receptor-1 (PD-1) is upregulated in many tumors and in their surrounding microenvironment, and blockade of these immune checkpoints with anti-PD-1 monoclonal antibodies has led to remarkable clinical responses in melanomas, non-small-cell lung cancer, renal-cell carcinoma, bladder cancer, and Hodgkin’s lymphoma [1–3]. High numbers of somatic mutations in lung cancer due to cigarette smoke and in melanoma due to ultraviolet radiation correlated with response to PD-1 blockade but not PD-1 expression [4]. Correlation of immune to the tumor mutation load was first noted with CTLA blockade in melanoma [5]. DNA mismatch repair machinery is essential in governing the genomic integrity, and loss of DNA mismatch repair function complex can occur either at the germ-line level or at the epigenetic level summarized elsewhere [6]. Mismatch repair plays a central role in maintaining genomic stability by repairing DNA replication errors and inhibiting recombination between non-identical (homologous) sequences [7]. Dr. Le and Diaz group conducted a pivotal phase II study on pembrolizumab (KEYTRUDA), an anti-programmed death 1 checkpoint inhibitor, in 41 patients with previously treated progressive metastatic carcinoma with or without mismatch repair deficiency. This phase 2 study administered pembrolizumab (10 mg/kg every 2 weeks). Three groups were evaluated: mismatch repair (MMR)-deficient colorectal cancer (*n* = 13), MMR-proficient colorectal cancer (*n* = 25), and MMR-deficient other cancers (*n* = 10). Mismatch repair status was assessed using a standard polymerase chain reaction (PCR)-based method for detection of microsatellite instability. The primary endpoints of the study were immune-related progression-free survival (PFS) rate as assessed at 20 weeks and overall response rate; secondary endpoints included overall survival (OS) and progression-free survival (as measured by RECIST v1.1) and disease control rate.

Despite the prior chemotherapy with some patients receiving up to four lines of chemotherapy, pembrolizumab resulted in response rate, progression-free survival, and overall survival for a diverse group of MMR-deficient cancers, but not in MMR-stable cancers [8]. The immune-related objective response rate and immune-related progression-free survival rate were 40 % (4 of 10 patients) and 78 % (7 of 9 patients), respectively, for mismatch repair-deficient colorectal cancers and 0 % (0 of 18 patients) and 11 % (2 of 18 patients), respectively, for mismatch repair-proficient colorectal cancers. The median progression-free survival and overall survival were not reached in the cohort with mismatch repair-deficient colorectal cancer but were 2.2 and 5.0 months, respectively, in the cohort with mismatch repair-proficient colorectal cancer (hazard ratio for disease progression or death, 0.10 (*P* < 0.001), and hazard ratio for death, 0.22 (*P* = 0.05)). Patients with mismatch repair-deficient non-colorectal cancer had responses similar to those of patients with mismatch repair-deficient colorectal cancer (immune-related objective response rate, 71 % (5 of 7 patients); immune-related progression-free survival rate, 67 % (4 of 6 patients)). Whole-exome sequencing revealed a mean of 1782 somatic mutations per tumor in mismatch repair-deficient tumors, as compared with 73 in mismatch repair-proficient tumors (*P* = 0.007), and high somatic mutation loads were associated with prolonged progression-free survival (*P* = 0.02). The most common treatment-related adverse events (occurring in greater than or equal to 10 % of patients) included rash/pruritus (17 %), pancreatitis (15 %), and thyroiditis/hypothyroidism (10 %). Grades 3–4 treatment-related adverse events were rare occurring in 2 % of patients (*N* = 1). This seminal work confirmed the earlier observation that only 1 of 33 patients with colorectal cancer with MMR deficiency responded to pembrolizumab and higher mutation loads (up to 20-fold) found in MMR-deficient tumors correlated with both immune-based response and improved progression-free survival to PD-1 blockade [8]. Increased mutations loads in the MMR-deficient tumors lead to increased neo-antigens which serve as a biomarker in cancer immunotherapy and as novel therapeutic tumor vaccine to selectively enhance T cell across different tumor types [9]. Membranous PDL-1 expressions were noted in all MMR-deficient tumors and correlate with the presence of CD8 cells but fail to predict in vivo immune response, progression, or survival in this small study. Based on this promising study, two studies sponsored by Merck were launched including KEYNOTE-164: a phase II study of pembrolizumab (MK-3475) as monotherapy in subjects with previously treated locally advanced unresectable or metastatic (stage IV) mismatched repair-deficient or microsatellite instability-high colorectal carcinoma (NCT02460198) and CheckMate 142 study of nivolumab and nivolumab plus ipilimumab in recurrent and metastatic colon cancer (NCT02060188).

Anti-PD-1 therapy will become the first immune therapy backbones for these MMR-deficient tumor patients whose median age is in their 40s as opposed to 60s in patients with MMR-proficient tumors [7, 10]. MMR deficiency is commonly found across a spectrum of cancers: uterus (~30 %), stomach (9 %), colorectum (15 %), ampulla of biliary tract (9 %) [11], acinar gland of the pancreas (14 %) [12], ovary (10–30 %) [13, 14], prostate (19 %) and small intestine (23 %) as well as in breast cancer (5 %) [15]. Mismatch repair-deficient tumor microenvironment strongly expressed several immune checkpoint ligands, including PD-1, PDL1, CTLA-4, LAG-3, and IDO, which indicates that their active immune microenvironment is counterbalanced by immune inhibitory signals that resist tumor elimination. As compared to MMR-stable tumors, MMR-deficient cancers of colorectum [16], small intestine [17], acinar gland of the pancreas [12], stomach, ampulla [11], prostate [18], and ovary [14], except for uterine cancer [19, 20], are associated with improved survival [21]. Increased immune surveillance in MMR deficiency in the tumors may in fact explain the pattern of earlier stage presentation, lack of lymph node metastasis (stage II in colon cancer), and restored immune suppression following primary tumor resection. MMR-deficient tumors enjoyed infiltration of cytotoxic T cells in both the tumor and the close border around the tumor, as well as increased intra-epithelial infiltration with higher angiogenic capacity [22]. A low intra-epithelial CD3(+)/FoxP3(+) cell ratio predicted reduced DFS 46.2 vs 66.7 % survival at 5 years [16].

MMR deficiency (or microsatellite instability) is noted in both hereditary tumors as well as in sporadic tumors due germ-line mutations or aberrant methylation of promoter region CpG islands in the MMR genes [7]. Genes upstream of regulating MSH2 degradation, i.e., FRAP1 (also known as MTOR) HERC1, PRKCZ, and PIK3C2B, can also lead to undetectable levels of MSH2 protein and MSI instability. Even though, RFC, PCNA, POLδ, and RPA are essential parts of DNA repair machinery and their defects could be lethal to living cells. However, POLδ *variant*, *POLε variant*, or *MYH* could also lead to MMR deficiency phenotype. Of MMR DNA repair complex, MLH1 and MSH2 are dominant players in safeguarding the genome from promiscuous recombination and their defect leads to complete loss of mismatch repair function whereas MSH6, MLH2, MSH3, and PMS1 are relatively redundant and exert weaker effects. MMR complex interacts with pivotal genes such as p53, c-Abl, and p73 regulating mismatch repair-dependent apoptosis pathway, transcriptional regulation, signaling transduction, DNA repair, immune surveillance, and drug resistance (Fig. 1) [6, 23–25]. Methylated p14 is associated with the presence of microsatellite instability and with the absence of p53 mutations. The impact of other genetic mutations on MMR could impact effects of chemotherapy as well as immune response. Mutations in both alleles of the hMLH1 gene are necessary for the manifestation of defective mismatch repair. There are 100 times more mutation loads in the MMR-deficient tumors than in the MSI-stable tumors. MSI phenotype testing by the current IHC or PCR methods may not reveal the full spectrum of high mutation load tumors suitable for therapy with anti-PD-1 blockade. Combining MSI testing and mutation load through next generation sequencing (NGS) may further expand the eligible patient pool for anti-PD-1-based therapy and multi-tumor basket trial.

**Fig. 1:**
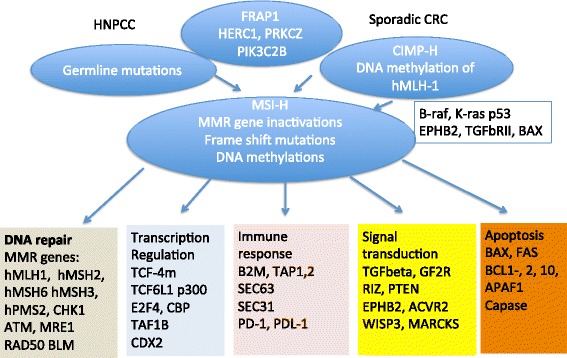
Microsatellite instability is central in colorectal cancer carcinogenesis in both hereditary nonpolyposis syndrome and sporadic colorectal cancer through germ-line mutations in MMR genes or by hMLH-1 DNA methylation in the CIMP-H, respectively. Microsatellite instability affects DNA repair, transcription regulation, signaling, and apoptosis

Pembrolizumab resulted in immune-based response in high mutation load MMR-deficient tumors and moderate overall survival than in MMS-stable tumors. However, the progression-free survival and overall survival gain remain modest in this small pilot study. To bring down the tumor giant, PD-1 may be one of the Achilles heels of tumor to target. Immune editing, clonal T cells repertoire deletions, and strong immunosuppressive microenvironment are some of the underlying mechanisms for non-responders to PD-1 blockade. There are complex interplay between the tumor, the supporting tumor microenvironment, and the immune system at both the local and systemic levels contributing to tumor regression as well as progression. Combination immune checkpoint inhibitors including PD-1, PDL-1 LAG-3, OX40, and IDO may provide additional boost in immune response against the tumor as well as increase in toxicities. In addition, PD-1 checkpoint inhibitor may be combined with tumor-specific T (CAR-T) and NK cells with or without dendritic cells and tumor vaccines for priming. Another approach is to suppress cytotoxic T suppressor and myeloid-derived suppressor cells (MDSC). Recently, we had witnessed a progress using unique structure of chimeric antigen receptor (CAR)-endows T cell tumor conferring specific cytotoxicity and resistance to immunosuppressive microenvironment in cancers. Challenges remain before widespread clinical application especially if one were to combine that with additional checkpoint inhibitors. Re-engineered NK cells may hold certain advantages over T cells, and synergy with PD-1/PDL-1 blockade will need to be explored also in the future [26]. One will also need to figure out how to combine or sequence standard first-line cytotoxic chemotherapy and/or targeted therapy with checkpoint inhibitor in the MMR-deficient tumors. Alkylating agent requires an intact DNA mismatch repair mechanism to cause DNA double-strand breaks to lead to tumor cell apoptosis and mediate certain resistance to cytotoxic agents [23–25]. Combine or sequence standard first-line cytotoxic chemotherapy and/or targeted therapy with checkpoint inhibitor in the MMR-deficient tumors. Both TS and DPD (5FU resistance genes) are overexpressed in MMR-deficient tumors as compared with MMR-proficient tumors explaining the relative drug resistance of MMR-deficient tumors to 5FU-based therapy. Mutations type II receptor for TGF-beta1 with high levels of microsatellite instability point to a favorable outcome to fluorouracil-based adjuvant regimens for stage II and stage III colon cancer [27]. B-raf mutation is linked to the MMR deficiency of these tumors in repairing mismatched bases in DNA and is associated with poor prognosis in colorectal cancer [28]. One direction is to explore B-raf inhibition in combination with immune checkpoint inhibition, but the sequencing and secondary pathways perturbed by B-raf mutation inhibition will need to be understood [29]. Other option is to explore MEK and inhibitor can also be explored in combination therapy [30]. For non-colorectal cancer, many of the tumor utilizes platinum based or 5FU-based regimen and integrating PD-1 blockade in these tumors will require careful understanding of the tumor response characteristics, sequencing, and tumor heterogeneity. Thus, first-line treatment with agents that deplete or inhibit key immune-suppressing stroma molecules and that provide co-stimulatory support, treatment using vaccines that induce an immune response in non-immunogenic cancers, or a combination of these agents should be the first step toward recruiting activated T cells into the tumor.

In summary, enhanced efficacies without systemic toxicities are the primary goals of anti-cancer therapy including immunotherapy. Major therapeutic gains have been made in a wide variety of solid tumors by blocking programmed death-1/programmed death ligand-1 (PD-1/PD-L1) pathways. The mechanisms of anti-PD-1/PDL-1 blockade differ from other immunotherapeutic approaches in that it leads to more tumor-specific immune modulation by targeting tumor-induced immune defects and by repairing ongoing tumor immunity instead of de novo immunity using other approaches, e.g., CAR-T. We will need more reliable biomarkers to predict, recognize, monitor, define, and grade immune-mediated adverse events as well as response across all tumor types and tissues and organs in light of the observation that we see increasing toxicities in GI, liver function, and endocrine abnormalities from treatment especially combination treatment strategies which is the way of the future [31]. Success of anti-PD-1/PDL1 has already set a very higher bar for future innovations to excel in more efficacious and less toxicities in all human tumors. Now, this tumor Goliath is in the open fields and PD-1 blockade represents its first successful shot at its Achilles heel; however, much of the work remains in order to bring down this tumor Goliath to its knees by targeting other unexposed Achilles heels and its healing mechanisms.
